# Identification of coffee bean varieties using hyperspectral imaging: influence of preprocessing methods and pixel-wise spectra analysis

**DOI:** 10.1038/s41598-018-20270-y

**Published:** 2018-02-01

**Authors:** Chu Zhang, Fei Liu, Yong He

**Affiliations:** College of Biosystems Engineering and Food Science, Zhejiang University, Hangzhou, 310058 China

## Abstract

Hyperspectral imaging was used to identify and to visualize the coffee bean varieties. Spectral preprocessing of pixel-wise spectra was conducted by different methods, including moving average smoothing (MA), wavelet transform (WT) and empirical mode decomposition (EMD). Meanwhile, spatial preprocessing of the gray-scale image at each wavelength was conducted by median filter (MF). Support vector machine (SVM) models using full sample average spectra and pixel-wise spectra, and the selected optimal wavelengths by second derivative spectra all achieved classification accuracy over 80%. Primarily, the SVM models using pixel-wise spectra were used to predict the sample average spectra, and these models obtained over 80% of the classification accuracy. Secondly, the SVM models using sample average spectra were used to predict pixel-wise spectra, but achieved with lower than 50% of classification accuracy. The results indicated that WT and EMD were suitable for pixel-wise spectra preprocessing. The use of pixel-wise spectra could extend the calibration set, and resulted in the good prediction results for pixel-wise spectra and sample average spectra. The overall results indicated the effectiveness of using spectral preprocessing and the adoption of pixel-wise spectra. The results provided an alternative way of data processing for applications of hyperspectral imaging in food industry.

## Introduction

Coffee is one of the most popular beverage in the world. Coffee variety is among the key factors influencing the coffee quality and price. According to International Coffee Organization (ICO), the estimated average number of global coffee consumption in the past 4 years was higher than 8 × 10^6^ tons^1^. Identification of coffee beans has been studied by traditional reagent-based laboratory chemical methods^2,3^, spectroscopy techniques^4,5^ and digital imaging techniques^6,7^. Reagent-based chemical methods are time consuming, reagent dependent and complex to operate. Spectroscopy and imaging techniques have been widely adopted as rapid, non-destructive and accurate techniques. Hyperspectral imaging (HSI), a technique integrating both spectroscopy and imaging techniques, has drawn raising attentions from researchers of different fields. HSI acquires spectral and spatial information simultaneously. The hyperspectral image is a three-dimensional (3D) data cube (x, y, λ) with the two-dimensional spatial information (x, y) and the third dimension of spectral information (λ). Each pixel has a spectrum in the hyperspectral image together with a gray-scale image at each wavelength. Hyperspectral imaging has been reported to detect coffee quality^8–13^, and the use of pixel-wise spectra have not been discussed in coffee quality determination.

One of the main advantages of hyperspectral imaging is to form and visualize the distribution maps of the samples. It reveals not only the physical attributes but also the chemical compositions within or between samples. Theoretically, visualization of the physical attributes and the chemical compositions by HSI is feasible and applicable. The wide applied procedure for image visualization is to apply the calibration models using sample average spectra to pixels within the samples. However, it is impossible to measure the physical attributes and chemical compositions of each pixel, the correctness of the prediction maps cannot be guaranteed. The recent studies have used the average prediction value or the trend of the changes of the physical attributes and chemical compositions of the samples^14–18^. But for classification issues, the category of each pixel within the sample are previously known (except mixtures). The accuracy of the prediction map could be testified for each pixel.

There are three major factors influencing the performances of the prediction maps. The first factor is that, the reference values of the physical attributes and chemical compositions are measured in terms of average values in the calibration set. The reference values of some parts within the sample (represented by pixels within the hyperspectral image) may be beyond the range of the reference values. Applying the calibration model to the pixels with physical attributes and chemical compositions beyond the range of the calibration set would result in inaccurate prediction values.

Another crucial factor which would lead to an inaccurate prediction is the uneven sample surface and shapes. For samples with irregular shape, the spectra of different parts would be different due to the different distance from the detector to these regions. The average of pixel-wise spectra reduces the effects of the sample shape. When predicting physical attributes and chemical compositions of pixels, the differences of pixel-wise spectra caused by sample shape should be considered. Considering that pixel-wise spectra within the samples would display the detailed sample information, the establishment of the calibration models using pixel-wise spectra could be an effective alternative for spectral data analysis in hyperspectral images. The use of pixel-wise spectra can significantly expand the number of samples and the range of sample features. Studies have been reported to conduct spectral data analysis based on pixel-wise spectra^19–23^.

Last but not least, noise is also a typical issue for using pixel-wise spectra to build calibration models. The average spectrum of a sample in a hyperspectral image is averaged by hundreds even more of the pixel-wise spectra. The random noises of the average spectra are significantly reduced, whereas the noises of the pixel-wise spectra are not reduced at all. Hence, the application of calibration models using average spectra to each pixel may result in inaccurate prediction values, which is also a critical reason for the inaccuracy of the prediction map. Spectral preprocessing is one of the most important steps to reduce the influence of noises and scattering. Different spectral preprocessing methods have different influences on the model performance. Generally a comparison is made to select the optimal preprocessing method^24,25^. Processing of average spectra and pixel-wise spectra have been used in spectral data analysis of hyperspectral imaging^19,21,23^. For hyperspectral images, the spectral preprocessing of each pixel would result in the changes of the reflectance value of each wavelength to reduce spectral noises. For the gray-scale image at each wavelength, spatial preprocessing can result in changes of gray value of each pixel, and gray value of each pixel is the reflectance value of the pixel at the wavelength. Thus the spatial preprocessing can also result in changes of reflectance value. Therefore, it is crucial to explore the influence of spectral and spatial preprocessing of hyperspectral images on spectral features and model performances.

The objective of this study was to explore the methods to reduce the random noises of hyperspectral imaging for better visualization of distribution map. Our specific objectives were to: (1) explore the influence of spatial and spectral preprocessing on the spectrum of a single pixel to reduce random noises; (2) explore the influence of spatial and spectral preprocessing on the discriminant models; (3) explore the differences between the pixel-wise spectra based discriminant models and the average spectra based discriminant models, and the prediction maps formed by the two kinds of models.

## Results

### Spectral profile

**Figure 1:**
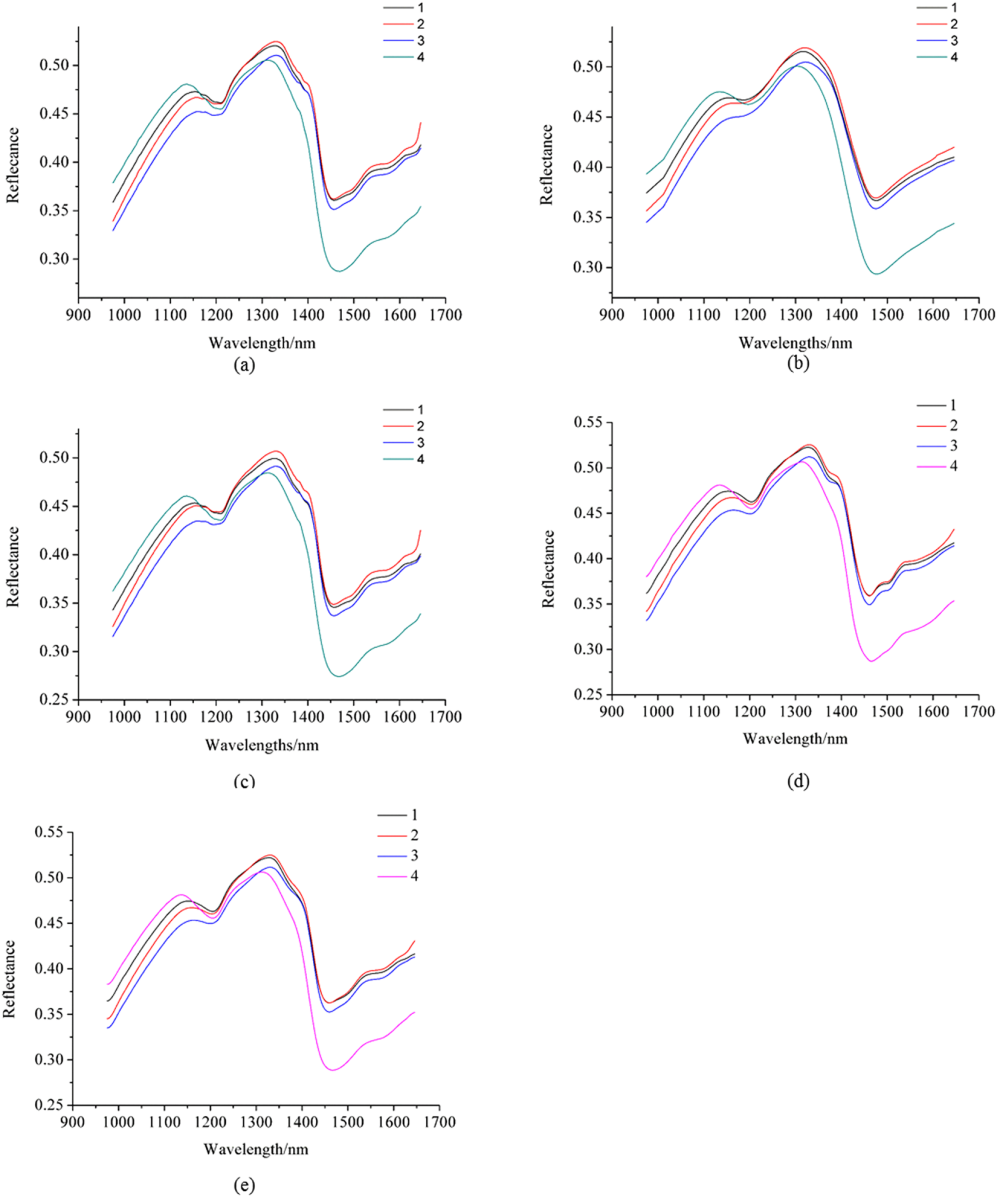
(**a**) Average spectra of unpreprocessed spectra; (**b**) average spectra extracted from the pixels preprocessed by MA with smoothing points of 23; (**c**) average spectra extracted from the image preprocessed by median filter with window size of 11 × 11; (**d**) average spectra extracted from the pixels preprocessed by WT; (**e**) average spectra extracted from the pixels preprocessed by EMD. MA7 means moving average smoothing with 7 points, MF3 means median filter with window size of 3 × 3, and similarly hereinafter.

The average spectra of the unpreprocessed hyperspectral images from the samples of the 4 coffee varieties are shown in Fig. 1(a). The number 1, 2, 3 and 4 were assigned as the category values of the 4 coffee varieties (Typic Arabica coffee from Yunnan Province as 1, Catimor Arabica coffee from Yunnan Province as 2, Fushan Robusta coffee from Hainan Province as 3 and Xinglong Robusta coffee from Hainan Province as 4). The average spectra of the samples of the 4 varieties with the pixel-wise spectra preprocessed by moving average (MA) with smoothing points of 23 are shown in Fig. 1(b). The average spectra of the samples of the 4 varieties extracted from the image preprocessed by median filter (MF) with window size of 11 × 11 are shown in Fig. 1(c). The average spectra of the samples of the 4 varieties with the pixel-wise spectra preprocessed by wavelet transform (WT) are shown in Fig. 1(d). The average spectra of the samples of the 4 varieties with the pixel-wise spectra preprocessed by empirical mode decomposition (EMD) are shown in Fig. 1(e).

It could be found that the average spectrum of each variety showed differences between each other with or without preprocessing. The average spectra in Fig. 1(b) showed smoother spectrum. Some reflectance peaks and valleys were missed, indicating the loss of information. The average spectra in Fig. 1(c) showed the same trends and features as the average spectra in Fig. 1(a). The average spectra in Fig. 1(c) showed lower reflectance value than those in Figs. 1(a) and 1(b). Figure 1(d) and (e) show similar spectra with Fig. 1(a).

**Figure 2:**
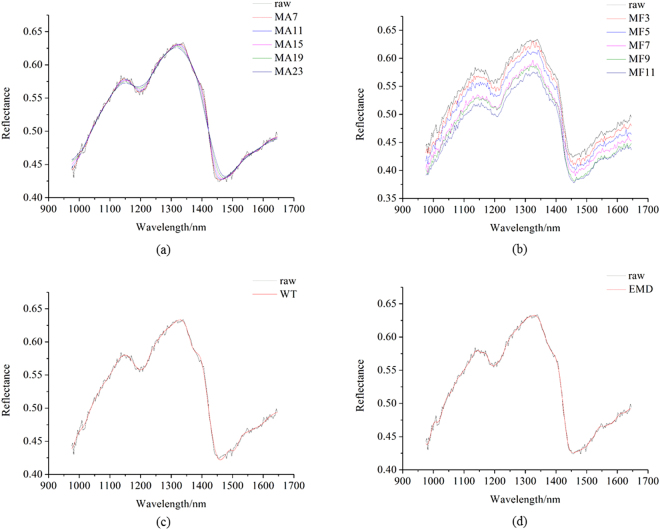
(**a**) Raw pixel-wise spectrum of a randomly selected pixel and the corresponding spectrum preprocessed by MA with different smoothing points; (**b**) raw pixel-wise spectrum and the corresponding spectrum preprocessed by MF with different window sizes; (**c**) raw pixel-wise spectrum and the corresponding spectrum preprocessed by WT; (**d**) raw pixel-wise spectrum and the corresponding spectrum preprocessed by EMD.

Figure 2(a) shows raw pixel-wise spectrum of a randomly selected pixel and the corresponding spectrum preprocessed by MA with different smoothing points. Figure 2(b) shows the raw pixel-wise spectrum and the corresponding spectrum preprocessed by MF with different window sizes. Figure 2(c) shows the raw pixel-wise spectrum and the corresponding spectrum preprocessed by WT. Figure 2(d) shows the raw pixel-wise spectrum and the corresponding spectrum preprocessed by EMD.

It could be found that the unpreprocessed spectrum of the single pixel showed similar spectral curve comparing with that of the average spectra. The blurs caused by random noises were obvious. As shown in Fig. 2(a), with the increasing smoothing points of MA, the spectrum became smoother. When the number of smoothing points reached to 15, 19 and 23, no obvious burrs could be observed, and some reflectance peaks and valleys disappeared. It indicated the loss of information. As shown in Fig. 2(b), the burrs were randomly distributed with different window size, and the reflectance value became lower with the increase of the filter window size. As shown in Fig. 2(c) and (d), the preprocessed spectrum showed smooth spectrum and no obvious blurs. Compared with Fig. 2(a) and (b), spectrum preprocessed by WT and EMD showed better fit with the original raw spectrum, with randomly distributed noises removed.

### Spilt of the calibration and prediction set

A total of three hundreds of coffee beans of each coffee variety were used for hyperspectral images acquisition and one image was taken from fifty coffee beans were acquired in one hyperspectral image. Four images of each variety of coffee beans were used as the calibration set (a total of 200 coffee beans of each variety).The remaining two images were used as the prediction set (a total of 100 coffee beans of each variety). Both average spectra and pixel-wise spectra for calibration and prediction were extracted from the coffee beans in the predefined calibration and prediction set.

### Classification models on full sample average spectra

The SVM models were built on full sample average spectra to evaluate the influence of spatial and spectral preprocessing on coffee bean variety identification. In this case, each coffee bean was used as an individual sample, and the average spectra of each sample were defined as sample average spectra. The classification results are shown in Table 1. The performances were evaluated by classification accuracy, defined as the percentage of correctly classified samples taken from all the samples.

**Table 1:** The SVM models using full sample average spectra.

	c	g	Cal (%)	CV (%)	Pre (%)
raw	147.033	3.031	99.750	97.750	97.250
MAS7	256.000	3.031	99.375	96.625	97.750
MAS11	256.000	5.278	99.125	95.250	95.750
MAS15	256.000	5.278	98.750	93.875	94.500
MAS19	256.000	1.741	95.875	93.125	93.750
MAS23	256.000	3.031	96.250	92.750	93.250
MF3	256.000	1.741	99.625	98.125	97.250
MF5	256.000	1.741	99.625	97.875	97.750
MF7	256.000	1.741	99.625	97.750	97.250
MF9	256.000	3.031	99.625	97.750	98.000
MF11	256.000	3.031	99.625	97.750	98.000
WT	256.000	3.031	99.500	97.250	98.250
EMD	256.000	3.031	99.500	97.000	98.000

The SVM models using full sample average spectra performed well with classification accuracy over 90% under different pretreatments. For pixel-wise spectra preprocessed by MA smoothing, the classification accuracy slightly decreased when the smoothing points increased. The reason might be that moving average smoothing not only reduced the noises but also reduced the useful information, especially when the smoothing points were large. The prediction accuracy was 93.250% for smoothing points of 23. For spectra extracted from the image preprocessed by MF, the classification results were similar to unpreprocessed spectra and better than spectra preprocessed by MA, and no specific regulation could be found. The reason might be that MF kept the useful information and did not reduce the noises, and the noises were only randomly redistributed with lower reflectance value. In general, the SVM models on the spectra extracted from preprocessed image by MF performed better than those by MA. Though the classification results for pixel-wise spectra preprocessed by WT and EMD were not the best. The sample average spectra based models showed good results with no significant differences. These results indicated that the preprocessing of pixel-wise spectra and images had little influence on sample average spectra based models.

### Classification models on full pixel-wise spectra

**Figure 3:**
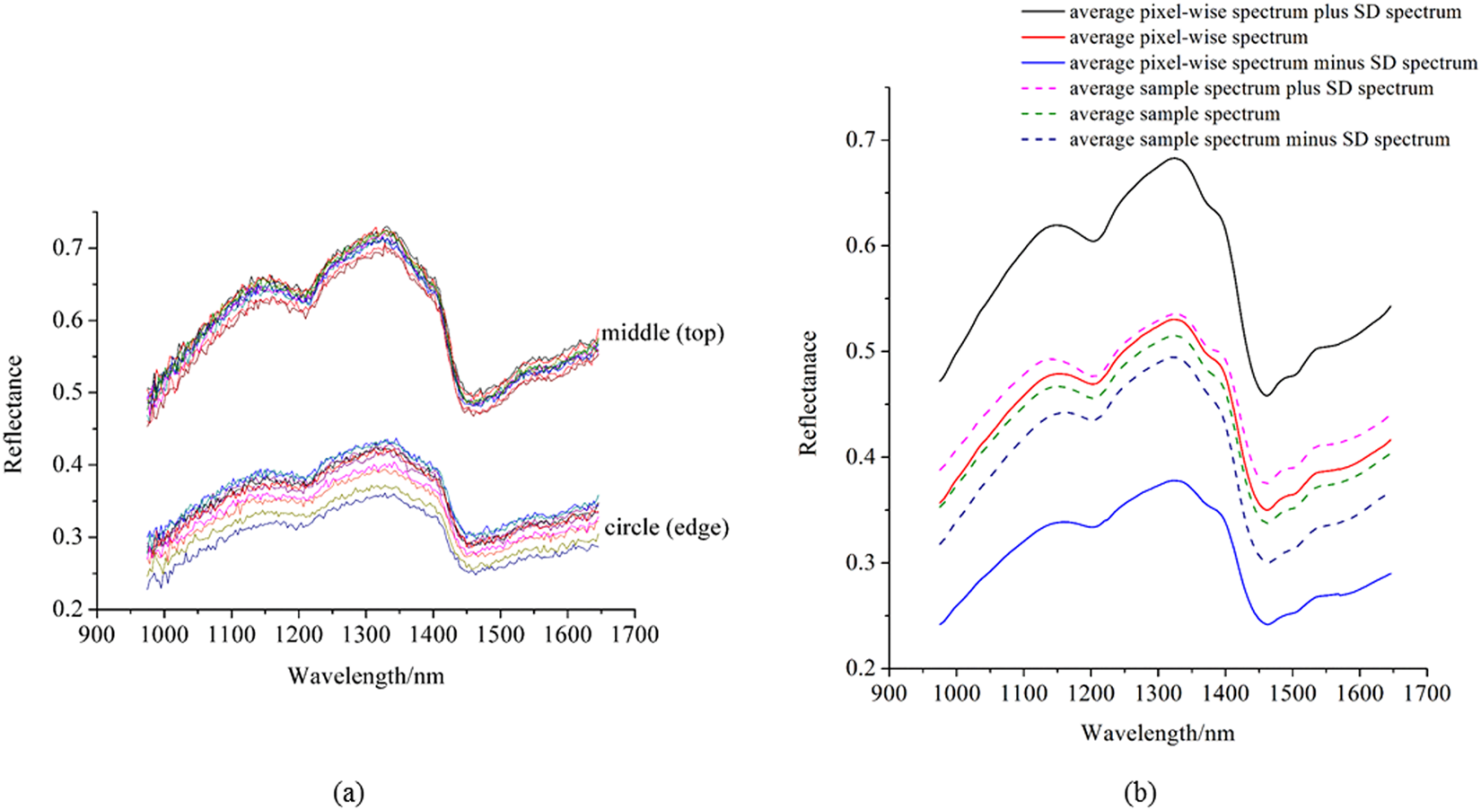
(**a**) The spectra of 10 randomly selected pixels in the middle and 10 randomly selected pixels in the outer race; (**b**) average spectrum, average spectrum plus standard deviation (SD) spectrum and average spectrum minus SD spectrum of calibration sets formed by pixel-wise spectra (solid lines) and sample average spectra (dash lines).

Preprocessed and unpreprocessed pixel-wise spectra were also used to build classification models. In this hyperspectral imaging system, the distances between different parts of the coffee beans and the detector were different, resulting in different reflectance intensity. The spectra of 10 randomly selected pixels in the middle part and 10 randomly selected pixels in the outer race are shown in Fig. 3(a). It could be found that the spectra of pixels in the middle had much higher reflectance values than those in the outer race, and the reflectance were beyond that of the average spectra shown in Fig. 1.

There were nearly 600,000 pixels in all 1200 coffee beans of the 4 varieties. Considering that WT and EMD could reduce random noises effectively, pixel-wise spectra preprocessed by WT and EMD were used to build discriminant models. Pixel-wise spectra for calibration were extracted from the 200 coffee beans in the calibration set. For each variety, 2000 pixels were randomly selected. Eight thousands of pixel-wise spectra were selected from over 404,854 pixel-wise spectra. Pixel-wise spectra for prediction were extracted from the 100 coffee beans in the prediction set, and the number of pixel-wise spectra in the prediction set was 200,554. The classification results are listed in Table 2. As shown in Table 2, the SVM models using pixel-wise spectra preprocessed by WT and EMD achieved good results, with classification accuracy for calibration and prediction over 80%. However, the classification results of SVM models using pixel-wise spectra were worse than those of SVM models using average spectra. The reason might be that pixel-wise spectra greatly extended the spectral features of coffee beans. It was possible that coffee beans form different varieties had similar quality attributes (such as chemical components), which may result in misclassification, and limited number of coffee beans (300 of each variety) failed to explore the possible similarity.

**Table 2:** The SVM models using full pixel-wise spectra.

	c	g	Cal (%)	CV (%)	Pre (%)
WT	256.000	0.574	89.938	87.413	90.887
EMD	256.000	0.574	88.013	85.138	88.426

### Optimal wavelengths selection

**Figure 4:**
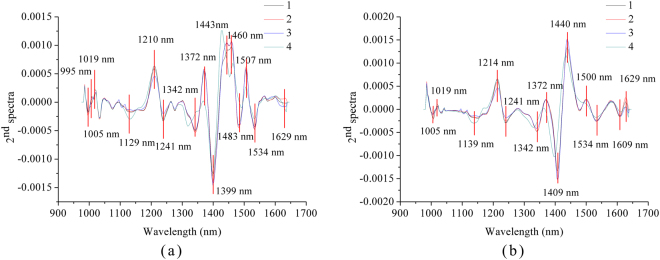
Optimal wavelengths selected by 2^nd^ spectra of WT (**a**) and EMD (**b**) preprocessed spectra.

Second derivative spectra (2^nd^ spectra) were used to select optimal wavelengths. To obtain 2^nd^ spectra, average spectra of each cultivar in the calibration set of sample average spectra were used. SVM models using pixel-wise spectra preprocessed by WT and EMD and the corresponding sample average spectra showed good classification results. Thus, second derivative spectra acquired by average spectra from pixel-wise spectra preprocessed by WT and EMD are shown in Fig. 4. The high peaks and low valleys could be identified from the second derivative spectra. As shown in Fig. 4, some peaks and valleys of different varieties of coffee bean showed quite small differences, which indicated the small differences in the corresponding wavelengths. These peaks and valleys were not selected as optimal wavelengths for coffee variety identification. The peaks and valleys with large differences within the second derivative spectra of four coffee varieties were manually selected as optimal wavelengths. Fifteen and thirteen optimal wavelengths were selected for spectra preprocessed by WT and EMD. The selected optimal wavelengths for spectra preprocessed by WT and EMD were quite similar but not the same, indicating that pixel-wise spectra preprocessing could influence the influence the selection of optimal wavelengths, and the influence was not significant.

The selected wavelength between 973 nm and 1020 nm (995, 1005 and 1019 nm) might be attributed to the second overtone of N-H stretch^26^. The wavelength between 1100–1300 nm (1129, 1139, 1210, 1214, 1241 nm) might be attributed to the second overtone of C-H stretch^27^. The wavelength between 1300 nm and 1400 nm (1342, 1372 and 1399 nm) might be ascribed to combination bands of C-H vibrations^27^. The wavelengths at 1409, 1440, and 1443 and 1460 nm might be ascribed to water bands^28^. The wavelength around 1480 nm (1483 nm) was attributed to the second overtone of O-H stretch^29^. The wavelength at 1500 nm was ascribed to the combination of CH2 stretching and nonstretching^30^. The wavelength at 1507 nm and 1534 nm might be attributed to the first overtone of N-H stretch^31^. The wavelength around 1608 nm (1609 nm) might be attributed to the first overtone of C-H stretch^32^. The wavelength around 1630 nm (1629 nm) was attributed to the aromatic C-H band^33^.

### Classification models on the optimal wavelengths

The classification results of the SVM models using optimal wavelengths from sample average spectra and pixel-wise spectra are shown in Table 3. The SVM models using the optimal wavelengths of sample average spectra performed well with classification accuracy over 90%, while SVM models using optimal wavelengths of pixel-wise spectra obtained worse results. The classification accuracies of SVM models using optimal wavelengths from pixel-wise spectra preprocessed by WT and the corresponding sample average spectra obtained better results than the models using optimal wavelengths from pixel-wise spectra preprocessed by EMD and the corresponding sample average spectra.

**Table 3:** The SVM models using the optimal wavelengths.

		c	g	Cal (%)	CV (%)	Pre (%)
Sample average spectra	WT	256.000	27.858	96.125	92.500	92.250
	EMD	256.000	48.503	97.625	94.500	94.000
Pixel-wise spectra	WT	256.000	16.000	86.013	82.650	80.482
	EMD	256.000	16.000	82.750	79.450	77.653

Comparing the results in Tables 1, 2 and 3, the SVM models using full spectra performed slightly better than the corresponding models on optimal wavelengths. The classification accuracy of the calibration and the cross validation were quite close in all models, while classification accuracy of the prediction were slightly worse. The overall results in Tables 1, 2 and 3 showed that full spectra and optimal wavelengths of sample average spectra and pixel-wise spectra could be used to identify coffee bean varieties. The preprocessing methods had influences on the classification results.

### Prediction results of pixel-wise spectra by sample average spectra based models and Prediction of sample average spectra by pixel-wise spectra based models

Pixel-wise spectra and sample average spectra all showed spectral features of coffee beans. Use of sample average spectra based models to predict the corresponding pixel-wise spectra in the prediction set (200554 pixels) and use of pixel-wise spectra based models to predict the corresponding sample average spectra in the prediction set (400 samples) were explored. The results of using full spectra and optimal wavelengths are shown in Table 4.

**Table 4:** Results of using pixel-wise spectra based models to predict sample average spectra and using sample average spectra based models to predict sample average spectra.

	Models	Prediction of pixels by models using sample average spectra		Prediction of samples by models using pixel-wise spectra	
		Cal (%)	Pre (%)	Cal (%)	Pre (%)
Full spectra	EMD	99.500	42.613	88.013	96.000
	WT	99.500	41.946	89.938	97.500
Optimal wavelength	EMD	97.625	38.645	82.730	87.250
	WT	96.125	39.562	86.013	88.500

It could be found that use of sample average spectra based models to predict the corresponding pixel-wise spectra obtained unsatisfactory results, with classification accuracy of prediction lower than 50%. Use of sample average spectra based models to predict the corresponding pixel-wise spectra obtained satisfactory results, with classification accuracy of prediction over 80%. The reason might be that the pixel-wise spectra covered more information of coffee beans, when use of sample average spectra based models to predict the corresponding pixel-wise spectra, some of spectral features were beyond the calibration set of sample average spectra. Figure 3(b) shows the average spectra of the calibration set of pixel-wise spectra, and the average spectra plus and minus the standard deviation spectra. The average spectra of the calibration set of sample average spectra, and the average spectra plus and minus the standard deviation spectra are also shown in Fig. 3(b). It could be found that pixel-wise spectra showed wider spectral features than sample average spectra. The results indicated that pixel-wise spectra could be used to identify coffee bean varieties.

### Prediction maps

Prediction maps were formed by using sample average spectra based model (using optimal wavelengths) to predict the corresponding pixel-wise spectra in the prediction set, and by using pixel-wise spectra based model (using optimal wavelengths) to predict the corresponding pixel-wise spectra in the prediction set. The prediction maps are shown in Fig. S1. As shown in Fig. S1 (in the supplementary file), significant differences could be observed from the prediction maps formed by sample average spectra and pixel-wise spectra. Prediction maps formed by pixel-wise spectra were much better. Each variety of coffee beans could be accurately identified. The prediction maps formed by sample average spectra misclassified mostly of coffee beans, except for one variety (Xinglong Robusta coffee from Hainan Province). Moreover, the different part of a coffee bean in the prediction maps formed by sample average spectra showed significant differences, indicating the influences of sample shape. Sample shape was an inevitable factor in prediction maps. It could be seen from the prediction maps that pixel-wise spectra were feasible to be used to build classification models and form prediction maps.

## Discussion

Hyperspectral imaging had the advantage of capturing spectral and spatial information simultaneously, and each pixel had a spectrum. The spectral and spatial preprocessing of hyperspectral images all resulted in the changes of pixel-wise spectra, and the corresponding sample average spectra (shown in Figs 1 and 2). According to Figs 1 and 2, MF could not reduce the random noises, but it changed the spectra of each pixel on spectral reflectance; MA could reduce the random noises, but useful information could be missed with the increase of smoothing points; WT and EMD could reduce the random noises, meanwhile keep the spectral profiles. However, the sample average spectra were averaged by hundreds or more of pixel-wise spectra, the average procedure could significantly reduce the random noises. Therefore, although pixel-wise spectra showed obvious differences, the SVM models using sample average spectra showed slight differences on classification results. The results indicated that sample average spectra could be used for coffee bean variety identification without preprocessing of pixel-wise spectra.

Pixel-wise spectra of samples within the hyperspectral images showed spectral features of different parts. Pixel-wise spectra could also be used to build discriminant models. Random noises should be firstly reduced from pixel-wise spectra. As shown in Fig. 3, due to the sample shape, the spectral features of pixel-wise spectra beyond the sample average spectra. Thus, prediction results of pixel-spectra by using sample average spectra based models showed unsatisfactory results, with classification accuracy lower than 50%. On the contrary, prediction results of sample average spectra by using pixel-wise spectra based models showed good results. The results indicated that pixel-wise spectra could be used to build discriminant models to predict both pixel-wise spectra and sample average spectra. Sample shape as a factor was ignored by many studies. However, as in this study, sample shape showed significant influence on the spectral features, especially on the spectral intensity. The prediction results of pixel-spectra by using sample average spectra based models also proved it. The pixel-wise spectra in this study for the calibration (8000) were selected from over 400,000 pixels and the prediction set had 200,554 pixels. As shown in Fig. S1, significant differences could be observed from pixel-spectra based prediction and sample average spectra based prediction, and sample shape was a critical factor in forming a prediction map by sample average spectra. Some other studies have also used pixel-wise spectra for the analysis in hyperspectral imaging. Lara *et al*.^19^ used over 40,000 pixel-wise spectra to monitoring spinach shelf life through packing film; Diezma *et al*.^20^ used 3600 pixel-wise spectra to evaluate spinach quality stored under different conditions; Williams and Kucheryavskiy^21^ used pixel-wise spectra to classify maize kernels based on different hardness level; Vermeulen *et al*.^22^ used pixel-wise spectra to identify ergot bodies in wheat flour; Zhang *et al*.^23^ used pixel-wise spectra to classify twine, paper and plastic in different cultivars of cotton lint. The above studies obtained satisfactory results, which proved the feasibility of using pixel-wise spectra in hyperspectral imaging. However, how to select minimum representative pixels covering the most useful information should be further studied for better classification.

Optimal wavelength selection was important in spectral data analysis. In this study, it takes over 10 hours to build SVM model using 8000 full pixel-wise spectra, and the time reduced to about 20 minutes by using optimal wavelengths (Computer hardware: CPU: Intel Core (TM) i7–4710HQ; RAM: 16 GB; Graphics card: NVIDIA GeForce GTX 860 M; 256 GB solid state disk). Once the models were built, the prediction time is reduced to less than 60 seconds with 200,544 pixel-wise spectra in the prediction set. According to Fig. 4, preprocessing of pixel-wise spectra could influence the selection of optimal wavelengths. The influence was slight with minor shift of selected wavelengths. The results indicated the effectiveness of optimal wavelength selection by 2^nd^ spectra.

In all, preprocessing of pixel-wise spectra was efficient of pixel-wise spectra based modeling and prediction. There was no need to conduct spectral spatial preprocessing of hyperspectral images for sample average spectra based modeling and prediction. Pixel-wise based modeling extended the spectral features of the calibration set, and how to select representative pixel-wise spectra was an important issue to be studied in our future studies. This study also provided guides for image visualization by hyperspectral imaging that samples for image acquisition considering the effect of sample shape and size, and it would be better to evaluate the noise status of pixel-wise spectra before applying sample average based model on pixel-wise spectra.

## Methods

### Sample Preparation

Four varieties of coffee beans in China (Typic Arabica coffee from Yunnan Province, Catimor Arabica coffee from Yunnan Province, Fushan Robusta coffee from Hainan Province and Xinglong Robusta coffee from Hainan Province) were collected. All coffee beans were medium toasted, and coffee bean varieties were assigned corresponding values of 1, 2, 3 and 4. Three hundred intact coffee beans of each variety were used to acquire hyperspectral images.

### Hyperspectral imaging system

The hyperspectral imaging system was set up in a laboratory. The system consisted of an ImSpector N17E imaging spectrograph (Spectral Imaging Ltd., Oulu, Finland), a Xeva 992 camera (Xenics Infrared Solutions, Leuven, Belgium) with a OLES22 lens (Spectral Imaging Ltd., Oulu, Finland), two symmetrically placed 150 W tungsten halogen lamps (2900 Lightsource, Illumination Technologies Inc., USA) and a conveyer belt (Isuzu Optics Corp., Taiwan, China) for sample motion. The image acquisition system was placed in a dark room. A data acquisition and preprocessing software (Xenics N17E, Isuzu Optics Corp., Taiwan, China) was used to control the system and analyze the images. The system acquired images in the spectral range of 874–1734 nm with the spectral resolution of 5 nm and the spatial resolution of 320*256 pixels.

### Hyperspectral image acquisition and correction

Coffee beans were placed on a black plate with quite low reflectance, and the coffee beans could be easily isolated from the background. Fifty coffee beans of one variety were placed in the conveyer belt for hyperspectral image acquisition. Six images were obtained for each variety. In this study, to acquire clear and non-deformable images, the exposure time of the camera, the height between the lens and the plate and the moving speed of the conveyer belt were adjusted to 3500 µs, 17.9 cm and 13.8 mm/s, respectively.

The acquired images should be corrected to be analyzed. The corrected image (*I*_*c*_) was calculated by using the raw hyperspectral image (*I*_*raw*_), white reference image (*I*_*white*_) and dark reference image (*I*_*dark*_) according to the following equation:

1 $${I}_{c}=\frac{{I}_{raw}-{I}_{dark}}{{I}_{white}-{I}_{dark}}$$

### Hyperspectral image preprocessing

The corrected hyperspectral images contained noises which could not be avoided completely, and the spectral and spatial preprocessing of the hyperspectral images were used to minimize the noises. One of the main advantages of HSI was to use the average value of spectrum of each pixel within the ROI as the spectrum of the sample. The spectrum of each pixel contained noises, and the average procedure could reduce the random noises. However, the random noises of each pixel existed, and it was important to preprocess the hyperspectral images. Considering that HSI provided spectral data at a spectral range and the gray-scale image at each wavelength, the preprocessing of hyperspectral images were conducted in two ways - preprocess each gray-scale image using the MF with different window size (3 × 3, 7 × 7, 11 × 11, 15 × 15), and preprocess the spectral of each pixel using MA with different smoothing points (7, 11, 15, 19, 23 points), WT and EMD. Before image preprocessing, the coffee beans were isolated from the background by applying the masks to set the reflectance of background as 0. A binary image was built as mask using the gray-scale image at 1200 nm. In the mask, the sample region was set as 1 and the background region was set as 0. The binary image was applied to the gray-scale images at different wavelengths to isolate the coffee beans from the background.

### Spectral data extraction

To obtain the unpreprocessed spectra, the spectrum of each pixel within the ROI was extracted, and the average spectrum of the ROI was calculated and used as the spectrum of the sample. To obtain the spectra preprocessed by MA, the extracted spectrum of each pixel was preprocessed by MA with different smoothing points, the preprocessed spectrum of each pixel were then averaged as the spectrum of the sample. To obtain the spectra from the image preprocessed by median filter, the gray-scale image at each wavelength were preprocessed by median filter with different window size, and then the spectrum of each pixel were extracted and averaged as the spectrum of the sample. To obtain the spectra preprocessed by WT and EMD, the extracted spectrum of each pixel was preprocessed by WT and EMD, the preprocessed spectrum of each pixel were then averaged as the spectrum of the sample. Before spectral data extraction, the masks were built to isolate the coffee beans from the background.

### Chemometric methods

#### Preprocessing methods

Wavelet transform (WT) is a widely used denoising method in spectral analysis. WT is similar to Fourier transform. It decomposes the original spectra into high frequency part and low frequency part. High frequency part contained the noise information, and threshold values are set to remove the noise information. Then the processed high frequency part and the low frequency part are reconstructed to from the preprocessed spectra^34,35^.

Empirical mode decomposition (EMD) is a widely used signal denoising method. It decompose the original spectra into independent instrinsic mode functions (IMFs) and the residuals. The idea of using EMD for denoising is similar to WT. The first few IMFS contained the noise information, and threshold values are set to remove the noise information. Then the processed IMFs, the remaining IMFs and the residuals are reconstructed to from the preprocessed spectra^36,37^.

#### Optimal wavelength selection

Second derivative spectra (2^nd^ spectra) can improve spectral resolution, suppress spectral noises, highlight spectral peaks and avoid overlapping peaks. In 2^nd^ spectra, background information is suppressed, and spectral peaks related to chemical compositions can be identified from background information. Peaks and valleys with large differences in 2^nd^ spectra of the different cultivars can be selected as optimal wavelengths to identify the differences between different cultivars^38,39^.

#### Discriminant model

Support vector machine is also a supervised discriminant method. The output of SVM is the integer. SVM has good generalization ability, it could deal with both linear and nonlinear data efficiently^40–42^. A brief introduction of SVM is presented as following.

For a brief introduction of SVM, two classes situations are used in this section, and it is easy to extend two-class issues to multi-class issue^34^. Given a dataset ***S*** = {***X***, ***Y***} with two classes, where ***X*** = {*x*_1_, *x*_2_, …, *x*_*m*_}is the independent variable matrix with *m* samples and *n* features, ***Y*** = {*y*_1_, *y*_2_, …, *y*_*m*_} is the class labels (dependent variable) of the corresponding ***X***, ***Y*** contains two class labels +1 and −1. SVM are designed for both separable case and inseparable case.

For separable case, a hyperplane is constructed as:

2 $$w\cdot {\boldsymbol{X}}+b=0$$

where *w* is the normal direction of the hyperplane and *b* is the bias of the hyperplane. To obtain good classification results, the hyperplane maximizes the margin border under the constraints:

3 $${{y}}_{{\rm{i}}}({w}\cdot {{x}}_{{i}}+{b})\ge 1\,\,\,\,\,{i}=1,2,\ldots ,{\rm{m}}$$

Thus, the margin width is equal to $\frac{2}{\Vert {\rm{w}}\Vert }$, and to maximize the margin, $\Vert {\rm{w}}\Vert$ should be minimized according to the following equation:

4 $${\rm{\min }}(\frac{1}{2}{\Vert {w}\Vert }^{2})$$

Equation (4) is under the constraint of Equation (3).

To solve the problem of Equation (4), the Lagrangian function is introduced and the primal Lagrangian function is:

5 $${L}({w},{b},{\alpha })=\frac{1}{2}{\Vert {w}\Vert }^{2}-\sum _{{i}={\rm{1}}}^{{m}}{{\alpha }}_{{i}}{{y}}_{{i}}({{x}}_{{i}}\cdot {w}+{b})+\sum _{{i}={\rm{1}}}^{{m}}{{\alpha }}_{{i}}$$

The constraint of Equation (5) is ${{\alpha }}_{{\rm{i}}}\ge 0$ (${{\rm{\alpha }}}_{{\rm{i}}}$ is the Lagrangian multipliers). Equation (5) can be transformed to its dual problem under the conditions of Karush-Kuhn-Tucker:

6 $${{L}}_{{d}}({w},{b},{\alpha })=\sum _{{i}={\rm{1}}}^{{m}}{{\alpha }}_{{i}}-\frac{{\rm{1}}}{{\rm{2}}}\sum _{{i},{j}=1}^{{m}}{{\alpha }}_{{i}}{{\alpha }}_{{j}}{{y}}_{{i}}{{y}}_{{j}}({{x}}_{{i}}\times {{x}}_{{j}})$$

The constraints of Equation (6) are:

7 $$\sum _{{i}={\rm{1}}}^{{m}}{{\alpha }}_{{i}}{{y}}_{{i}}={\rm{0}}$$

8 $${\rm{w}}=\sum _{{i}=1}^{{m}}{{\alpha }}_{{i}}{{y}}_{{i}}{{x}}_{{i}}$$

The conditions of Karush-Kuhn-Tucker can be described as:

9 $${{\alpha }}_{{i}}[{{y}}_{{i}}({{x}}_{{i}}\cdot {w}+{b})-1]=0$$

After the optimization of *w* and *b*, the optimal hyperplane can be obtained and used for prediction. The classification function can be expressed as:

10 $${f}({x})={\rm{sign}}(\sum _{{\rm{i}}=1}^{{{n}}_{{sv}}}{\alpha }{iyi}({{x}}^{{{n}}_{{sv}}}\cdot {x})+{b})$$

where ${{n}}_{{SV}}$ is the number of support vectors.

For non-separable data, the positive slack variables ***ξ*** = {*ξ*_1_, *ξ*_2_, …, *ξ*_*n*_}are introduced, and the problem becomes minimize the following equation:

11 $$\frac{1}{2}{\Vert {\rm{w}}\Vert }^{2}+{C}\sum _{{\rm{i}}=1}^{{\rm{m}}}{\rm{\xi }}i$$

where *C* is the user-defined parameter, and *C* is used to assign penalty to errors. The constraints become:

12 $${{y}}_{{i}}({w}\cdot {{x}}_{{i}}+{b})-1+{{\xi }}_{{i}}\ge 0\,{\rm{with}}\,{{\xi }}_{{i}}\ge 0$$

To dear with non-separable data, the original data is mapped into a high dimensional feature space through non-linear mapping, and the attempt to construct a hyperplane which can deal with linear classification. Given ${\rm{\phi }}(X)$ as the high dimensional feature space mapped by ***X***, the Equation (6) can be written as:

13 $${{L}}_{{d}}({w},{b},{\alpha })=\sum _{{i}={\rm{1}}}^{{m}}{{\alpha }}_{{i}}-\,\frac{1}{2}\sum _{{i},{j}={\rm{1}}}^{{m}}{{\alpha }}_{{i}}{{\alpha }}_{{j}}{{y}}_{{i}}{{y}}_{{j}}({\rm{\phi }}({{x}}_{{i}}){\rm{\phi }}({{x}}_{{j}}))$$

In general, ${\rm{\phi }}({{x}}_{{i}}){\rm{\phi }}({{x}}_{{j}})$ is defined as kernel function. Kernel function is the key to map original data into new feature spaces. The used kernel function in this study is radial basis function (RBF), and it can be expressed as:

14 $${K}({{x}}_{{i}},{{x}}_{{\rm{j}}})=\exp (-\frac{\Vert {{x}}_{{i}}-{{x}}_{{\rm{j}}}\Vert }{2{{\rm{\sigma }}}^{2}})$$

where ${\rm{\sigma }}$ is tuning parameter referring to the bandwidth. The solutions to maximize Equation (13) can be reduced to Equation (7) and (8), and the conditions of Karush-Kuhn-Tucker become:

15 $${{\alpha }}_{{i}}\{{{y}}_{{i}}({w}\cdot {{x}}_{{i}}+{b})-1+{{\xi }}_{{i}}\}=0$$

where $0\le {{\alpha }}_{{i}}\le {C}$. Then the classification function can be expressed as:

16 $${f}({x})={\rm{sign}}(\sum _{{i}=1}^{{{\rm{n}}}_{{\rm{sv}}}}{\alpha }\mathrm{iyiK}({x},{xi})+{b})$$

where ${{n}}_{{SV}}$ is the number of support vectors.

## Electronic supplementary material

Supplementary Information
