# Supplementary material for: Identification of coffee bean varieties using hyperspectral imaging: influence of preprocessing methods and pixel-wise spectra analysis
Source: Sci Rep. 2018 Feb 1;8:2166. doi: 10.1038/s41598-018-20270-y (PMC5794930; doi:10.1038/s41598-018-20270-y)
Supplement: Supplementary file 1 — Supplementary Information [file 41598_2018_20270_MOESM1_ESM.pdf]

Identification of coffee bean varieties using hyperspectral imaging: influence of preprocessing methods and pixel-wise spectra analysis

Chu Zhang, Fei Liu*, Yong He*

College of Biosystems Engineering and Food Science, Zhejiang University, Hangzhou, 310038, China

*Corresponding author: Fei Liu and Yong He, E-mail: fliu@zju.edu.cn, yhe@zju.edu.cn, Tel./Fax.:+86-571-88982143

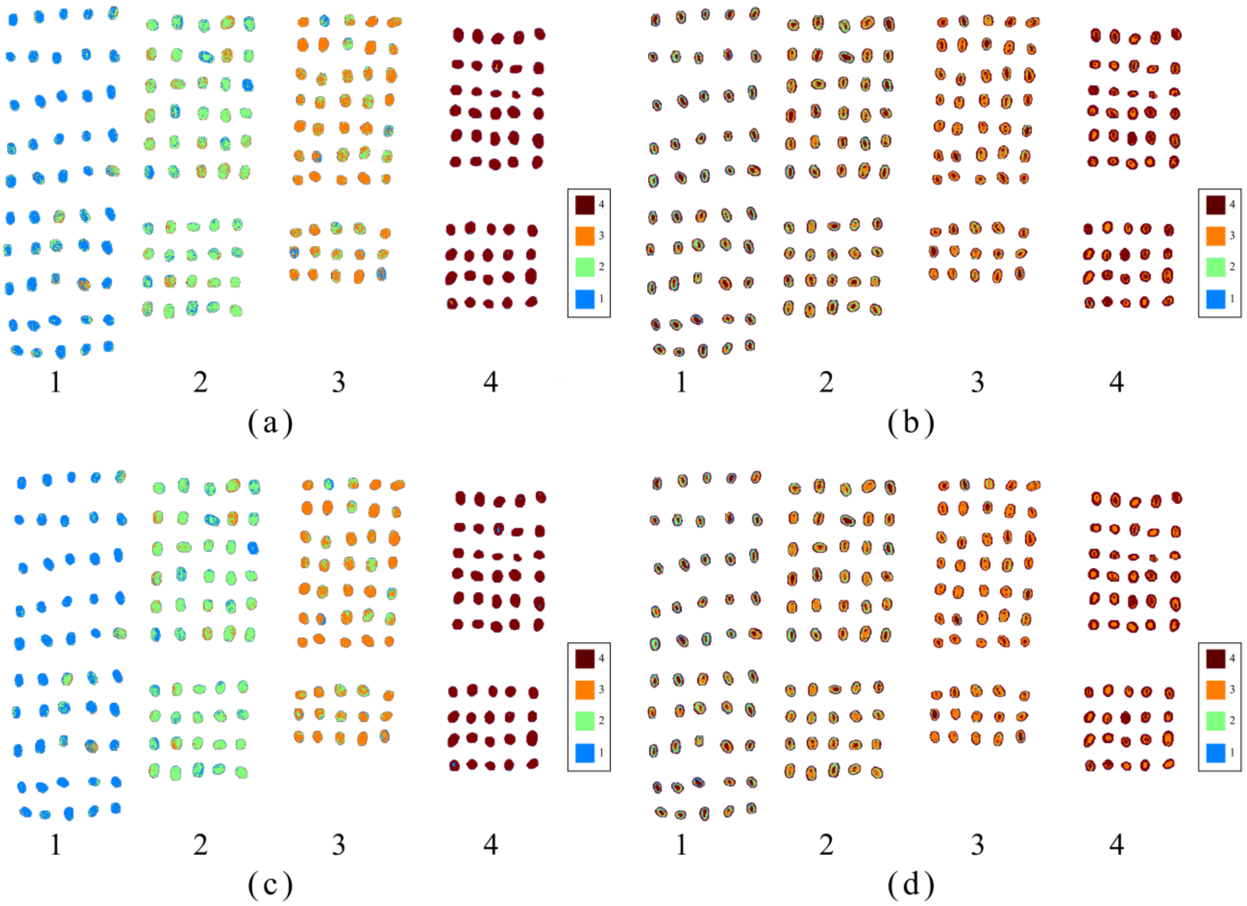

Fig. S1 Prediction maps of coffee beans: (a) prediction map of 4 varieties of coffee beans by applying SVM model built using optimal wavelengths selected from pixel-wise spectra (preprocessed by WT) to hyperspectral images; (b) prediction map of 4 varieties of coffee beans by applying SVM model built using optimal wavelengths selected from sample average spectra averaged by pixel-wise spectra (preprocessed by WT) to hyperspectral images; (c) prediction map of 4 varieties of coffee beans by applying SVM model built using optimal wavelengths selected from pixel-wise spectra (preprocessed by EMD) to hyperspectral images; (d) prediction map of 4 varieties of coffee beans by applying SVM model built using optimal wavelengths selected from sample average spectra averaged by pixel-wise spectra (preprocessed by EMD) to hyperspectral images.
